# Draft genome sequence of a kombucha isolate of *Pichia kudriavzevii*

**DOI:** 10.1128/mra.00478-25

**Published:** 2025-08-11

**Authors:** Steve A. James, Nicolle Som, Adrian Turner, David J. Baker, Arjan Narbad

**Affiliations:** 1 Food, Microbiome and Health Programme, Quadram Institute Bioscience 7308, Norwich, United Kingdom; 2 Centre for Microbial Interactions, Quadram Institute Bioscience 7308, Norwich, United Kingdom; 3 Microbes in the Food Chain, Quadram Institute Bioscience 7308, Norwich, United Kingdom; 4 National Collection of Yeast Cultures, Quadram Institute Bioscience 7308, Norwich, United Kingdom; University of Strathclyde, Glasgow, United Kingdom

**Keywords:** fungi, yeasts, *Pichia kudriavzevii*, *Candida krusei*, fermented foods, probiotics, genome sequencing

## Abstract

*Pichia kudriavzevii* (syn. *Candida krusei*) is a fermentative yeast of importance to both the food and biotechnology industries, as well as being of significant clinical concern. Here, we report the draft genome sequence of *P. kudriavzevii* NCYC 4488, a kombucha-derived isolate.

## ANNOUNCEMENT

*Pichia kudriavzevii* (syn. *Candida krusei*) is an ascomycetous yeast widely distributed in nature (1). Often found in spontaneous fermentations, it is used to produce a variety of traditional fermented drinks and beverages (1, 2), is a candidate probiotic (3), and has gained an increasing role in biotechnology (4, 5). Furthermore, in a clinical setting, it represents a notable opportunistic human pathogen (6, 7). Here, we combined short- and long-read sequencing to assemble the genome sequence of *P. kudriavzevii* NCYC 4488, isolated from a UK-produced organic kombucha. A 10 mL sample was 10-fold concentrated by centrifugation (10 mins and 4,000 rpm), plated onto Brain Heart Infusion (BHI) agar and incubated for 2 days at 30°C. Colonies were picked and purified by two further rounds of re-streaking on fresh BHI agar (incubated at 30°C). Colony isolate NCYC 4488 was identified as *P. kudriavzevii* by LSU D1/D2 sequencing using primers NL1 and NL4 (8). The D1/D2 sequence was deposited in GenBank (accession number PV526257.1).

Genomic DNA was isolated using a MasterPure yeast DNA purification kit (Cambio) with additional zymolyase and proteinase K treatment steps from a 2-day liquid Sabouraud dextrose culture grown at 30°C. A short-read library was prepared using a Nextera DNA Flex library prep kit (Illumina) with 20-fold diluted library reagents. Sequencing was performed on a NextSeq 500 sequencer, producing 7,997,848 paired-end 150 bp reads (~111× coverage). A Nanopore library was prepared from the same gDNA prep, without shearing or size selection, using the 1D ligation kit (catalog number SQK-LSK109, Oxford Nanopore Technologies, ONT), and loaded onto a FLO-MIN106 R9.4.1 flow cell (ONT) on a MinION instrument (ONT). This produced a total of 778,218 reads with an average read length of 4,687 bases (~337× coverage). Default parameters were used for all software unless otherwise specified. Base calling was performed using Guppy (ONT; v.3.6.0) in high-accuracy mode (model dna_r9.4.1_450bps_hac). Raw short- and long-read polishing was performed using fastp 0.23.2 (9).

*De novo* assembly with Nanopore reads was performed using Flye 2.9.1 (10), using long reads in “ONT regular reads, pre-Guppy5” mode. The assembly was polished with the Illumina data using three rounds of alignment with BWA-MEM v.0.7.17.1 (11) and Pilon v.1.24 (12) correction. Assembly statistics were generated using QUAST v.5.0.2 (13). The assembly consists of 10 contigs, including 4 chromosome-sized contigs, each terminating in either a 28 bp telomeric repeat sequence (5′-TTACAATATGAACTAGGAGCGAGGTGTG-3′)_n_ (14), identified using Tandem Repeats Finder v.4.09 (15), or an rDNA array (detected by barrnap v.0.9) (16). Minimap v.2.28 (17) was used to align these to the reference assembly (CBS 573; GenBank assembly GCA_003054445.1) and determine they corresponded to chromosomes 1, 2, 3, and 5 (14). The total length is 10,881,549 bp, with an N_50_ value of 2,747,939 bp, and a G + C content of 38.40%. Genome completeness was estimated as 96.2% using BUSCO v.5.5.0 (18) with the saccharomycete_odb10 data set. Augustus v.3.2.3 (19) predicted 5,063 protein-coding genes using the *Saccharomyces cerevisiae* training data set, and 175 tRNA genes were detected using tRNAscan SE 2.0 (20).

## Data Availability

This whole-genome shotgun project has been deposited in DDJB/ENA/GenBank (BioProject number PRJNA1251030, assembly accession number JBNDHO000000000.1). The version described in this paper is version 1. The raw reads are available in the NCBI Sequence Read Archive (SRA) under accession numbers SRX28403477 (ONT) and SRX28403476 (Illumina). The predicted protein and tRNA annotation data sets have been deposited in Zenodo (https://doi.org/10.5281/zenodo.16269560).
